# Histochemical quantification of collagen content in articular cartilage

**DOI:** 10.1371/journal.pone.0224839

**Published:** 2019-11-07

**Authors:** Lassi Rieppo, Lauriane Janssen, Krista Rahunen, Petri Lehenkari, Mikko A. J. Finnilä, Simo Saarakkala

**Affiliations:** 1 Research Unit of Medical Imaging, Physics and Technology, Faculty of Medicine, University of Oulu, Oulu, Finland; 2 Microelectronics Research Unit, Faculty of Information Technology and Electrical Engineering, University of Oulu, Oulu, Finland; 3 Department of Surgery and Intensive Care, Oulu University Hospital, Oulu, Finland; 4 Cancer and Translational Medicine Research Unit, Faculty of Medicine, University of Oulu, Oulu, Finland; 5 Infotech Oulu, University of Oulu, Oulu, Finland; 6 Department of Diagnostic Radiology, Oulu University Hospital, Oulu, Finland; Chung-Ang University College of Engineering, REPUBLIC OF KOREA

## Abstract

**Background:**

Articular cartilage (AC) is mainly composed of water, type II collagen, proteoglycans (PGs) and chondrocytes. The amount of PGs in AC is routinely quantified with digital densitometry (DD) from Safranin O-stained sections, but it is unclear whether similar method could be used for collagens.

**Objective:**

The aim of this study was to clarify whether collagens can be quantified from histological AC sections using DD.

**Material and methods:**

Sixteen human AC samples were stained with Masson’s trichrome or Picrosirius red. Optical densities of histological stains were compared to two commonly used collagen parameters (amide I and collagen CH_2_ side chain peak at 1338cm^-1^) measured using Fourier Transform Infrared (FTIR) spectroscopic imaging.

**Results:**

Optical density of Modified Masson’s trichrome staining, which included enzymatic removal of PGs before staining, correlated significantly with FTIR-derived collagen parameters at almost all depths of cartilage. The other studied staining protocols displayed significant correlations with the reference parameters at only few depth layers.

**Conclusions:**

Based on our findings, modified Masson’s trichrome staining protocol is suitable for quantification of AC collagen content. Enzymatic removal of PGs prior to staining is critical as us allows better staining of the collagen. Further optimization of staining protocols may improve the results in the future studies.

## Introduction

Articular cartilage (AC) provides a near frictionless surface in the joints during locomotion and redistributes the forces applied to bone ends[1]. In order to achieve these complex biomechanical properties, extracellular macromolecules require a proper structure and distribution. In osteoarthritis (OA), degenerative changes are observed in the AC, altering the structure, distributions, and amount of these macromolecules.

The main components of AC are water, collagens, proteoglycans (PGs) and non-collagenous proteins. From these components, collagens and PGs are often quantified in OA studies. PGs can be quantified spectrophotometrically by dimethyl methylene blue (DMMB) dye[2], which binds to negatively charged glycosaminoglycans. Collagens, on the other hand, are routinely quantified using high-pressure liquid chromatography[3] or biochemical assays, such as hydroxyproline quantification[4,5]. The major drawback of these quantification methods is their destructive nature and their inability to provide information about spatial distribution of the components unless spatially separate blocks are prepared from the sample.

Fourier Transform Infrared (FTIR) spectroscopic imaging can be used to analyze the biochemical composition of tissues from histological sections. Furthermore, it is also possible to determine the distributions of biochemical components within the sections with a spatial resolution of few micrometers. FTIR spectroscopic imaging has clear advantages over traditional biochemical methods. However, the FTIR microscopes are still relatively expensive and not available in all laboratories.

The morphological and compositional changes of AC are traditionally characterized using histochemical staining of different AC constituents. Histological evaluation of PG content is conducted using cationic stains, e.g., Safranin O[6], Alcian blue[7,8] or Toluidine blue[7,8], while stains for collagen include Masson’s trichrome[7,8] and Picrosirius red[7,8]. Histological stains are most often used only for qualitative evaluation of PG and collagen contents. However, the amount of PG in AC can be quantified from histological sections with digital densitometry (DD) as Safranin O binds stoichiometrically to the sulfated glycosaminoglycans of PGs[6,9]. DD can be conducted using regular light microscopes by obtaining a monochromator suitable for quantification of the studied stain and neutral density filters for calibration purposes. Therefore, DD method is simple and inexpensive, and can be taken in use practically in any laboratory using existing light microscopes.

In principle, DD could also be applied to histological sections stained with collagen specific dyes. However, it is unclear whether they bind stoichiometrically to collagens. The aim of this study was to clarify whether traditional collagen stains, such as Masson’s trichrome or Picrosirius red, are suitable for the quantification of the collagen content in histological sections of AC. DD was used to measure optical density of the used collagen stains. In addition, polarized light microscopy (PLM) was used to measure the retardance of unstained sections and Picrosirius red-stained sections.

## Materials and methods

### Samples

The study was approved by the ethical committee of the Northern Ostrobothnia hospital district (approval number 191/2000). Informed consent was obtained from all patients. Human AC samples (n = 16) were collected from patients (*N* = 12) undergoing total knee arthroplasty at Oulu University Hospital. Cylindrical osteochondral plugs were drilled from tibial plateaus of each tibia. Two plugs were prepared from four of the tibiae and one plug from the rest. Thus, sixteen osteochondral cores were used in the study. Subsequently, the plugs were fixed in formalin, decalcified in ethylenediaminetetraacetic acid (EDTA), dehydrated and embedded in paraffin for histological evaluation.

### Picrosirius red staining

Sections (thickness = 3 μm) were cut and placed on standard microscopy slides, deparaffined and rehydrated. Removal of PGs has been earlier shown to improve the staining results for Picrosirius Red[10]. To further investigate this result, Picrosirius red staining was done with and without enzymatic removal of PGs. In order to evaluate the effect of the removal of PG on the Picrosirius red stain, staining was performed with and without digesting PGs using papain (Acros Organics, 0.5% papain, 0.05 M phosphate buffer, pH = 4.4) at 37°C for 18 hours, followed by hyaluronidase treatment (Sigma Aldrich, 1000 U/ml in 0.1 M phosphate buffer, pH = 6.9) at 37°C for 18 hours. All tissue sections were rinsed with phosphate buffer and immersed in 0.1% direct red 80 (Sigma-Aldrich) in saturated picric acid for 60 minutes. Samples were then washed with 0.01M hydrochloric acid, dehydrated and mounted.

### Masson’s trichrome staining

Masson’s trichrome staining was conducted using ready–to-use kit (Trichrome Stain (Masson) Kit, HT15, Sigma-Aldrich). Briefly, the tissue sections (thickness = 5 μm) were cut and placed on standard microscopy slides. After deparaffinisation and rehydration, the slides were immersed in Bouin’s solution (HT 10132, Sigma-Aldrich) at 56°C for 15 minutes. Subsequently, the slides were washed with tap water for 5 minutes. Next, the sections were stained in Weigert’s hematoxylin for 5 minutes, and then washed again with tap water for 5 minutes and rinsed in distilled water. Next, the slides were stained in Biebrich scarlet-acid fuchsin for 5 minutes, rinsed in distilled water, incubated in phosphotungstic-phosphomolybdic acid for 5 minutes, dyed with aniline blue for 5 minutes, and fixed in 1% acetic acid for 2 minutes. Finally, the slides were rinsed in distilled water, dehydrated and mounted.

### Modified Masson’s trichrome staining

The Modified Masson’s trichrome staining was also conducted using ready–to-use kit (Trichrome Stain (Masson) Kit, HT15, Sigma-Aldrich), but the PGs were removed enzymatically in a similar way as in the Picrosirius red staining. Briefly, the tissue sections (thickness = 5 μm) were cut and placed on standard microscopy slides. After deparaffinisation and rehydration, the sections were digested with papain and hyaluronidase (0.5% papain, 0.05 M phosphate buffer, pH = 4.4 at 37°C for 18 hours followed by hyaluronidase (1000 U/ml in 0.1 M phosphate buffer, pH = 6.9) at 37°C for 18 hours). Sections were then immersed in Bouin’s solution (HT 10132, Sigma-Aldrich); stained in Weigert’s hematoxylin, incubated in phosphotungstic-phosphomolybdic acid, dyed with aniline blue and fixed in 1% acetic acid. Then, the slides were rinsed in distilled water, dehydrated and mounted. The Biebrich scarlet-acid fuchsin of the kit was not used, as it is not related to collagen, and may hinder the quantitation of the collagen stain. Otherwise, the procedure and staining durations were the same as described in previous section.

### Fourier Transform Infrared (FTIR) spectroscopic imaging

For the FTIR spectroscopic imaging measurements, 5-μm-thick sections were cut with a microtome (Thermo Scientific, Microm HM 355S) and transferred to microscope slides. Paraffin was dissolved with xylene before transferring the sections to 0.5-mm-thick Zinc-Selenide (ZnSe) windows (Crystran Ltd., Poole, United Kingdom). FTIR spectroscopic imaging measurements were conducted in transmission mode with a FTIR spectrometer (Tensor 27, Bruker Inc., Billerica, MA, USA) coupled with Bruker Hyperion 3000 microscope (Bruker Inc., Billerica, MA, USA) that is equipped with a focal plane array (FPA) 64x64 detector. The sample chamber was purged with dry air to minimize the atmospheric conditions. A rectangular region extending from the cartilage surface to subchondral bone was measured from each section. The spectral resolution was set to 4 cm^-1^ and each spectrum was averaged on 16 scans. Pixel binning (2x2) was used to set the pixel size to be 5.4 μm (native pixel size of the system is 2.7 μm). Noise was further reduced from the spectra by using a principal component analysis (PCA)-based noise filter (data was reconstructed using 20 first principal components). The integrated absorbance of the amide I (1585–1720 cm^-1^) peak was used to estimate the collagen content in AC. Furthermore, the absolute height of the collagen CH_2_ side chain vibration[11] at 1338 cm^-1^ was calculated from the 2^nd^ derivative spectrum to confirm the amide I peak results. Second derivative spectra were calculated using Savitzky-Golay algorithm with 9 smoothing points. Absolute value of the second derivative peak was used (2^nd^ derivative displays the absorbance peaks of original spectrum as negative peaks). All preprocessing and analysis of spectral data was performed with custom-made MATLAB (MathWorks Inc., MA, USA) scripts.

### Digital densitometry

DD was used to quantify the amount of stain in the sections[6]. DD measurements were conducted using the Carl Zeiss Axio Scope A1 microscope (Carl Zeiss, Germany) and QImaging QICAM camera (QImaging, Surry, BC, Canada). Sections were analyzed with monochromatic light at 625 nm for Masson’s trichrome and 550 nm for Picrosirius red. The measured grayscale values were calibrated to optical density (OD) using neutral density filters (range: 0–3 OD units, Edmund Optics, Blackwood, NJ, USA). A 1-mm-wide region-of-interest extending from the cartilage surface to cartilage-bone interface was used to analyze the images.

### Polarized light microscopy (PLM)

Similarly to previous studies, birefringence of collagen measured with polarized light microscopy (PLM) was used to quantitate the collagen content with[12,13] and without[12,14,15] Picrosirius red staining. The unstained sections (thickness = 5 μm) were placed on standard microscope slides, and the removal of PGs was done for these sections similarly as described earlier. The sections were imaged with Abrio PLM system (CRi, Inc., Woburn, MA, USA) mounted on a conventional light microscope (Nikon Diaphot TMD, Nikon, Inc., Shinagawa, Tokyo, Japan). The Abrio system consists of a green bandpass filter, a circular polarizer, and a computer-controlled analyzer composed of two liquid crystal polarizers, and a CCD camera. The system can be used for automated measurement of the magnitude of retardance in birefringent materials such as tissue sections. A 1-mm-wide region-of-interest extending from the cartilage surface to cartilage-bone interface was used to analyze the images.

### Data analysis and statistics

Regions of interests (ROIs) extending from the cartilage surface to cartilage-bone interface were manually selected from all (DD, PLM and FTIR spectroscopic imaging) images. Values on rows of the selected ROIs were averaged to obtain the mean value for different depths of the tissue. These depth-dependent profiles were scaled to 100 points to enable direct comparison between the methods. Comparisons were done in two ways: (i) One-tailed Pearson’s correlation analysis was conducted at all layers (1–100%) of rescaled depth-dependent DD or PLM profiles and FTIR spectroscopic imaging collagen profiles, and (ii) Pearson’s correlation coefficients were calculated sample-by-sample between the depth-dependent DD or PLM profiles and FTIR spectroscopic imaging collagen profiles. All statistical analyses were performed with (MathWorks Inc., MA, USA).

## Results

**Fig 1:**
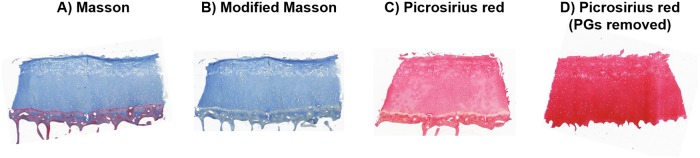
A cartilage sample stained using different collagen staining protocols. A) Masson’s trichrome, B) Modified Masson, C) Picrosirius red, and D) Picrosirius red after enzymatic removal of PGs.

Examples of a cartilage sample stained using the different collagen staining protocols are shown in Fig 1. Masson (Fig 1A) and Modified Masson (Fig 1B) look similar, expect for the lack of red color in Modified Masson since the Biebrich scarlet-acid fuchsin was not used. In case of Picrosirius red, the removal of PGs before staining (Fig 1D) significantly improved the staining compared to the Picrosirius red protocol in which PGs were not removed before staining (Fig 1C).

### Digital densitometry of Masson’s trichrome

**Fig 2:**
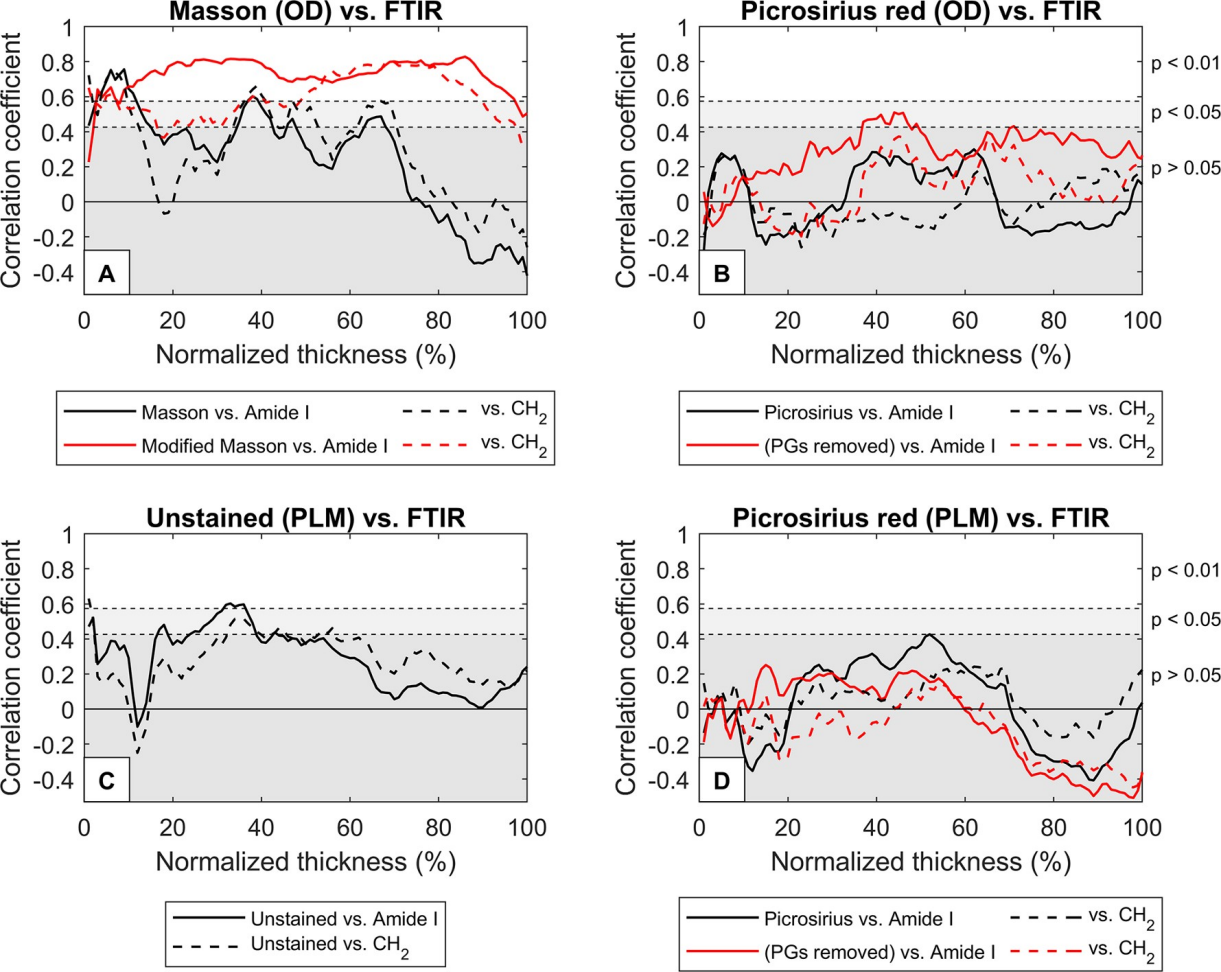
Correlations between the DD or PLM and FTIR-derived collagen contents at different layers of cartilage. A) Correlations between the optical density of Masson’s trichrome (black lines) or Modified Masson’s stain (red lines) and amide I absorbance (solid lines) or CH_2_ absorbance (dashed lines) at different layers. B) Correlations between the optical density of Picrosirius red without removal of PGs (black lines) or Picrosirius red after removal of PGs (red lines) and amide I absorbance (solid lines) or CH_2_ absorbance (dashed lines) at different layers. C) Correlations between the retardance of Picrosirius red without removal of PGs (black lines), Picrosirius red after removal of PGs (red lines) and amide I absorbance (solid lines) or CH_2_ absorbance (dashed lines) at different layers. D) Correlations between the retardance of unstained sections (black lines) and amide I absorbance (solid line) or CH_2_ absorbance (dashed line) at different layers.

**Fig 3:**
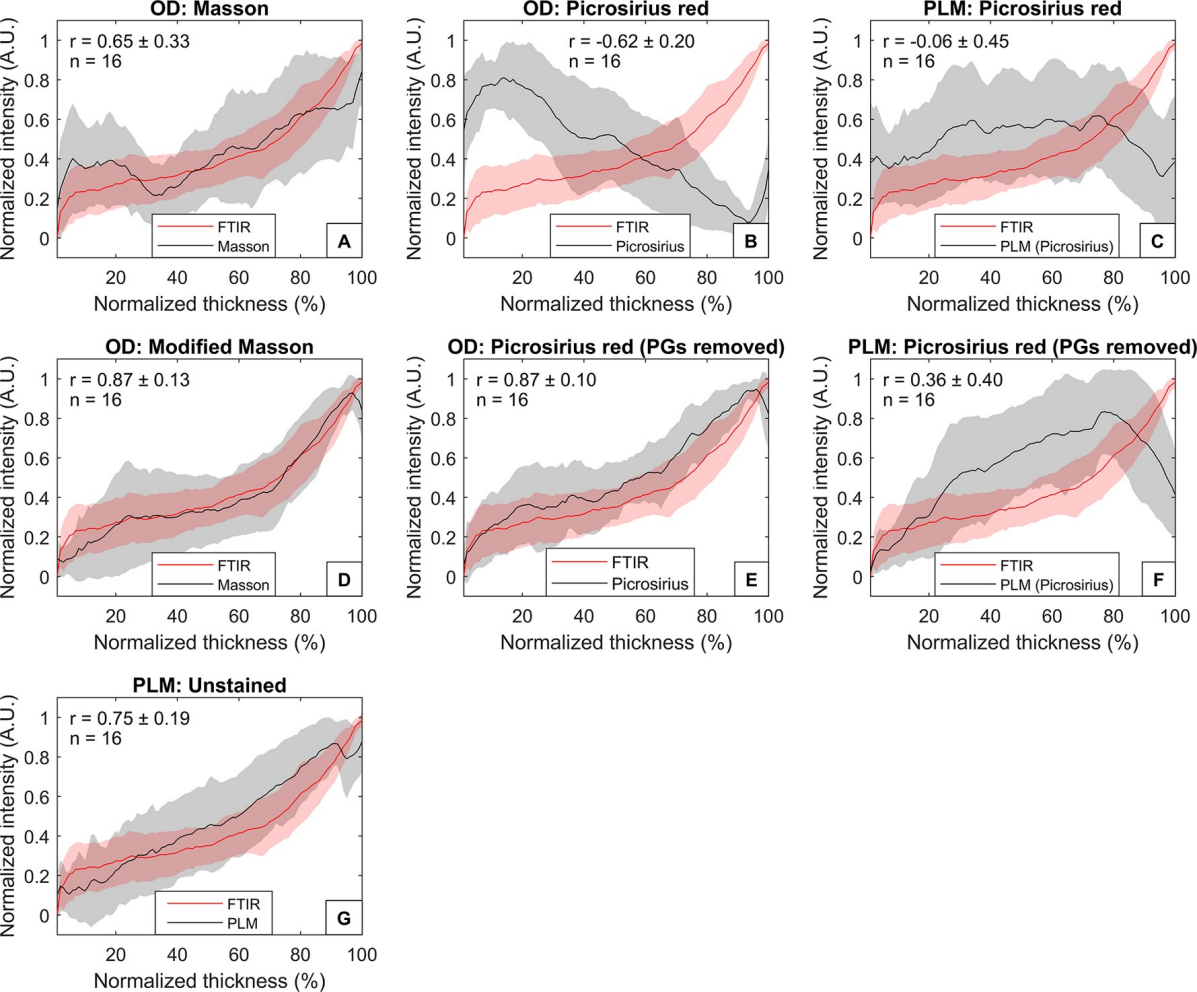
Depth-dependent average profiles of the collagen content compared with the amide I absorbance. The collagen content estimated by FTIR imaging (Amide I, red line) and A) the optical density of Masson’s trichrome, B) optical density of Modified Masson’s staining, C) optical density of Picrosirius red, D) optical density of Picrosirius red after removal of PGs, E) retardance of Picrosirius red-staining, F) retardance of Picrosirius red-staining after removal of PGs, and G) retardance of unstained sections. Shaded areas indicate the standard deviations of the sample set.

**Fig 4:**
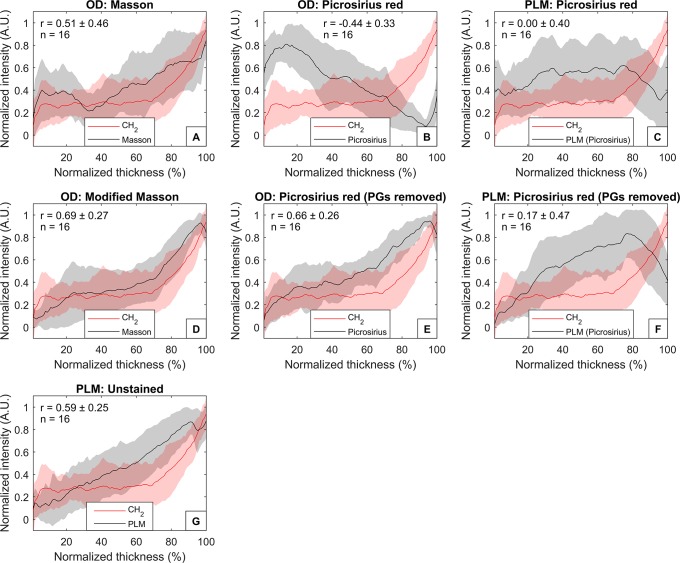
Depth-dependent average profiles of the collagen content compared with the collagen CH_2_ side chain vibration. The collagen content estimated by FTIR imaging (CH_2_, red line) and A) the optical density of Masson’s trichrome, B) optical density of Modified Masson’s staining, C) optical density of Picrosirius red, D) optical density of Picrosirius red after removal of PGs, E) retardance of Picrosirius red-staining, F) retardance of Picrosirius red-staining after removal of PGs, and G) retardance of unstained sections. Shaded areas indicate the standard deviations of the sample set.

Collagen quantification was performed based on the DD results at 625 nm of Aniline Blue of Masson’s Trichrome stained tissue slides with and without removal of PGs. Without removal of PGs, significant correlations (r > 0.426) were observed at superficial layer and some parts of the deep layer, but the mean correlation (± standard deviation) was low with both the amide I (r = 0.25 ± 0.31) and CH_2_ absorbances (r = 0.29 ± 0.27) (Fig 2A). On average, sample-by-sample correlations between the depth-dependent profiles of optical density of Masson’s staining and the amide I absorbance were strong (r = 0.65 ± 0.33) (Fig 3A), and slightly weaker in case of CH_2_ absorbance (r = 0.51 ± 0.46) (Fig 4A).

The modified Masson protocol improved the correlations: significant correlation was observed with amide I in all but the most superficial (1–2%) layer (mean correlation: r = 0.72 ± 0.10) (Fig 2A). The average correlations between the modified Masson and amide I depth-dependent profiles were also strong (r = 0.87 ± 0.13) (Fig 3D). Significant correlations were also observed with CH_2_ absorbance in all layers except a portion of middle layer (17–20%) and the deepest layer (98–100%) of cartilage (mean correlation: r = 0.59 ± 0.13) (Fig 2A). Furthermore, the sample-by-sample correlations with the CH_2_ absorbance profiles were strong (r = 0.69 ± 0.27) (Fig 4D). Summaries of the correlation analyses are seen in Tables 1 and 2.

**Table 1:** Correlations between the densitometry and polarized light microscopy methods and amide I absorbance.

	Correlations coefficients at different layers (1–100%)			Correlation coefficients between depth-dependent profiles (n = 16)		
	Mean correlation	Standard deviation	Min–Max	Mean correlation	Standard deviation	Min–Max
**OD**						
Masson	0.25	0.31	-0.42–0.76	0.65	0.33	-0.25–0.97
Modified Masson	0.72	0.10	0.23–0.83	0.87	0.13	0.53–0.97
Picrosirius red	0.02	0.18	-0.28–0.30	-0.62	0.20	-0.87–-0.25
Picrosirius red (PGs removed)	0.29	0.14	-0.14–0.51	0.87	0.10	0.61–0.96
**PLM**						
Picrosirius red	0.02	0.25	-0.41–0.43	-0.06	0.45	-0.83–0.71
Picrosirius red (PGs removed)	-0.07	0.25	-0.51–0.25	0.36	0.40	-0.56–0.92
Unstained	0.28	0.18	-0.10–0.60	0.75	0.19	0.34–0.96

The correlation analysis was conducted at each depth layer (1–100%) of cartilage. In addition, depth-dependent profiles were compared sample-by-sample using correlation analysis.

**Table 2:** Correlations between the densitometry and polarized light microscopy methods and CH_2_ absorbance.

	Correlations coefficients at different layers (1–100%)			Correlation coefficients between depth-dependent profiles (n = 16)		
	Mean correlation	Standard deviation	Min–Max	Mean correlation	Standard deviation	Min–Max
**OD**						
Masson	0.29	0.27	-0.26–0.74	0.51	0.46	-0.86–0.86
Modified Masson	0.59	0.13	0.31–0.80	0.69	0.27	0.20–0.96
Picrosirius red	-0.01	0.13	-0.26–0.27	-0.44	0.33	-0.88–0.40
Picrosirius red (PGs removed)	0.09	0.15	-0.20–0.37	0.66	0.26	0.06–0.97
**PLM**						
Picrosirius red	0.05	0.12	-0.20–0.24	0.00	0.40	-0.60–0.65
Picrosirius red (PGs removed)	0.13	0.16	-0.45–0.15	0.17	0.47	-0.75–0.73
Unstained	0.28	0.15	-0.25–0.63	0.59	0.25	0.00–0.89

The correlation analysis was conducted at each depth layer (1–100%) of cartilage. In addition, depth-dependent profiles were compared sample-by-sample using correlation analysis.

### Digital densitometry of picrosirius red

No significant correlation was found between the optical density of Picrosirius red and amide I or CH_2_ at any layer when PGs were not removed before the staining (Fig 2B). Furthermore, the average correlations between the depth-dependent profiles of the optical density of Picrosirius red and the FTIR spectroscopic collagen parameters (amide I and CH_2_) were negative (Figs 3B and 4B).

Removal of PGs enhanced the staining of deep cartilage and, thus, improved the mean correlations of optical density of Picrosirius red with amide I and CH_2_ absorbances. However, significant correlations were found only between the optical density of Picrosirius red and amide I at some layers of deep cartilage (37–47%, 49%, and 71%) (Fig 2B). No significant correlation was observed between the optical density of Picrosirius red (after removal of PGs) and CH_2_ absorbance (Fig 2B). Sample-by-sample correlations between the depth-dependent profiles of Picrosirius red (after removal of PGs) and amide I were strong (r = 0.87 ± 0.13) (Fig 3E), and slightly weaker with the CH_2_ (r = 0.66 ± 0.26) (Fig 4E).

### Polarized light microscopy

The retardance of Picrosirius red-stained sections with or without enzymatic removal of PGs displayed no correlation with the amide I or CH_2_ absorbances (except in one case: there was a significant correlation between the retardance of Picrosirius red and amide I at layer 52%) (Fig 2C). Furthermore, the depth-dependent profiles of the retardance of Picrosirius red-stained sections did not correlate with the depth-dependent profiles of amide I (Fig 3C and 3F) or CH_2_ (Fig 4C and 4F) absorbances.

The retardance of unstained AC sections showed a significant correlation with the amide I absorbance at certain depths (1–2%, 17–18%, 24–38%, 43%), but the mean correlation was low (r = 0.28 ± 0.18) (Fig 2C). Nevertheless, the correlations between the depth-dependent profiles were strong (r = 0.75 ± 0.19) (Fig 3G). Similarly, some significant correlations were observed between the retardance of unstained sections and CH_2_ absorbance (1–2%, 33–39%, 43–45%, 53–56%) (Fig 2C). On average, the correlations between the depth-dependent profiles of retardance of unstained sections and CH_2_ absorbance were moderate (r = 0.59 ± 0.25) (Fig 4G).

## Discussion and conclusions

Current collagen quantifications methods are based of destructive methods such as HPLC[3] or colorimetric hydroxyproline quantification[4,5]. A major drawback of these methods is that they do not allow the evaluation of the spatial distribution of collagen within the sample. In the current study, we showed that the optical density of Masson’s trichrome staining correlated with the collagen contents on histological sections after enzymatic removal of PGs, allowing the use of this technique for spatial quantification of the collagen content in histological slides. Optical density or retardance of the other studied stain, Picrosirius red, did not correlate with the reference collagen information at almost any depth of the tissue. Nevertheless, the depth-dependent profiles of both stains (after removal of PGs) correlated with the reference collagen profiles, suggesting that both stains reflect the collagen distribution within sections.

Our study compared the optical densities of Masson’s trichrome or Picrosirius red-stained histological sections with FTIR spectroscopic collagen parameters. Our findings suggest that densitometric measurement of histological sections stained with modified Masson’s trichrome produces information that is comparable to collagen distributions obtained using FTIR spectroscopic imaging. Enzymatic removal of PGs prior to staining was observed to be essential as it enhances the staining of collagen especially at deep cartilage. Exclusion of Biebrich scarlet-acid fuchsin from the modified Masson staining protocol may also have enhanced the specificity for collagen, although overall the effect is likely small, as the stain (seen as red/purple) was observed only in the calcified cartilage and bone (Fig 1A). Interestingly, even though the optical density of Picrosirius red did not correlate with the FTIR collagen parameters (except at some particular layers), the optical density of Picrosirius red (after removal of PGs) produced similar depth-dependent distributions as modified Masson’s staining and FTIR spectroscopic collagen parameters, as evidenced by the high correlation coefficients between the depth-dependent profiles. This may indicate that even though there is lots of variation in Picrosirius red staining between the samples, it is still suitable for evaluating the collagen distribution pattern within the sections.

These findings show that staining reflecting the collagen contents can be obtained by modified Masson’s Trichrome allowing spatial quantification of collagen in AC, in a similar way as the staining of PGs by Safranin O[6,9]. The use of phosphotungstic and phosphomolybdic acid as mordant during the Masson Trichrome staining enables a specific binding to the anionic NH3^+^ groups of the collagens at low pH, as pH few proteins remain acidic at low pH[16,17]. The retained phototungstic and phosphomolybdic acids then bind to Aniline blue cationic radicals allowing a specific binding to collagen[16,17]. This specific staining allows the quantification of collagen by DD. Although it was previously stated that the selectivity of collagen stains highly depends of the saturation of binding sites of non-collagen tissues with counterstaining[17], we observed that replacing the traditional Biebrich Scarlet-Fuchsin staining of the Masson’s Trichrome protocol by the removal of PGs by enzymatical digestion also allows specific binding of phototungstic and phosphomolybdic acids/aniline blue to collagens in the sections.

All DD and PLM data were compared with FTIR spectroscopic imaging data from an adjacent slide, using firstly the amide I absorbance (1585–1720 cm^-1^) of the FTIR spectra, and then the CH_2_ collagen side chain 1338 cm^-1^ absorbance. Although amide I absorbance is often used for quantification of cartilage collagen content, it may not be fully specific for collagens as glycosaminoglycans also have some absorbance in the same region[18]. On the other hand, the CH_2_ absorbance has been shown to be specific for collagens [11,18]. When the results of the amide I are compared with those of CH_2_, it seems that the amide I overestimates the collagen content within the deep cartilage at 50–70% of tissue depth. In general, DD and PLM results correlated more strongly with the amide I absorbance than with the CH_2_ absorbance, which suggests that the relationship between the amount of the studied stains and the collagen contents is not fully stoichiometric. Nevertheless, the modified Masson’s staining protocol correlated significantly with both collagen parameters practically at all tissue depths and can be viewed as the best option for densitometric quantification of collagen in AC. In the future, a study for comparing the DD results with the standard collagen quantifying methods, such as HPLC or colorimetric quantifications, should be conducted to further validate the DD quantification of the collagen content.

In general, the retardance of unstained sections or Picrosirius red-stained sections did not correlate with the collagen reference information. However, there was moderate or strong correlation between the depth-dependent profiles of retardance of unstained sections and FTIR collagen parameters. The collagen content is one factor that affects the magnitude of retardation, but there are other significant factors, as well. The anisotropy of collagen fibrils substantially affects the retardance [19]. Only a very weak retardance signal is seen in the transitional layer where the collagen fibrils are randomly oriented. On the other hand, a strong signal is seen at superficial layer (of healthy cartilage) and deep layers as the collagen fibrils are oriented in the same direction. Therefore, retardance images do not reflect only the collagen content, and, thus, should be interpreted very carefully. Picrosirius red is sometimes used for enhancing the retardance signal in PLM measurements [8,10,12,13,17]. In this study, the depth-dependent profiles of Picrosirius red-stained sections showed weak or no correlation with the collagen parameters. Based on our results, unstained sections are more suitable for PLM of AC than Picrosirius red-stained sections.

In conclusion, this new quantitative histological method for collagen staining allows spatial quantification of the collagen distribution in the whole section which is difficult or even impossible by standard biochemical analysis methods[3–5]. The histological quantification also opens new perspectives for less destructive collagen quantification, compared to previously used HPLC and hydroxyproline biochemical analysis[3–5]. Using histological slides instead of large tissue pieces for the quantification decreases the required number of samples to obtain statistically significant results and allows the comparison of a same sample with various techniques requiring histological sections such as FTIR spectroscopic imaging, PLM, histology, and immunohistochemistry.
